# Therapeutic Antibody-Like Immunoconjugates against Tissue Factor with the Potential to Treat Angiogenesis-Dependent as Well as Macrophage-Associated Human Diseases

**DOI:** 10.3390/antib7010008

**Published:** 2018-01-23

**Authors:** Zhiwei Hu

**Affiliations:** Department of Surgery Division of Surgical Oncology, The James Comprehensive Cancer Center, The Ohio State University College of Medicine, Columbus, OH 43210, USA; zhiwei.hu@osumc.edu; Tel.: +1-614-685-4606

**Keywords:** tissue factor, factor VII, antibodies, antibody-like immunoconjugates (ICON and L-ICON1), solid cancer, Leukemia, age-related macular degeneration, endometriosis, rheumatoid arthritis, atherosclerosis, angiogenesis, vascular endothelial cell, cancer cell, cancer stem cell, macrophage, fibroblast, B cell

## Abstract

Accumulating evidence suggests that tissue factor (TF) is selectively expressed in pathological angiogenesis-dependent as well as macrophage-associated human diseases. Pathological angiogenesis, the formation of neovasculature, is involved in many clinically significant human diseases, notably cancer, age-related macular degeneration (AMD), endometriosis and rheumatoid arthritis (RA). Macrophage is involved in the progression of a variety of human diseases, such as atherosclerosis and viral infections (human immunodeficiency virus, HIV and Ebola). It is well documented that TF is selectively expressed on angiogenic vascular endothelial cells (VECs) in these pathological angiogenesis-dependent human diseases and on disease-associated macrophages. Under physiology condition, TF is not expressed by quiescent VECs and monocytes but is solely restricted on some cells (such as pericytes) that are located outside of blood circulation and the inner layer of blood vessel walls. Here, we summarize TF expression on angiogenic VECs, macrophages and other diseased cell types in these human diseases. In cancer, for example, the cancer cells also overexpress TF in solid cancers and leukemia. Moreover, our group recently reported that TF is also expressed by cancer-initiating stem cells (CSCs) and can serve as a novel oncotarget for eradication of CSCs without drug resistance. Furthermore, we review and discuss two generations of TF-targeting therapeutic antibody-like immunoconjugates (ICON and L-ICON1) and antibody-drug conjugates that are currently being tested in preclinical and clinical studies for the treatment of some of these human diseases. If efficacy and safety are proven in current and future clinical trials, TF-targeting immunoconjugates may provide novel therapeutic approaches with potential to broadly impact the treatment regimen of these significant angiogenesis-dependent, as well as macrophage-associated, human diseases.

## 1. Introduction

**Figure 1:**
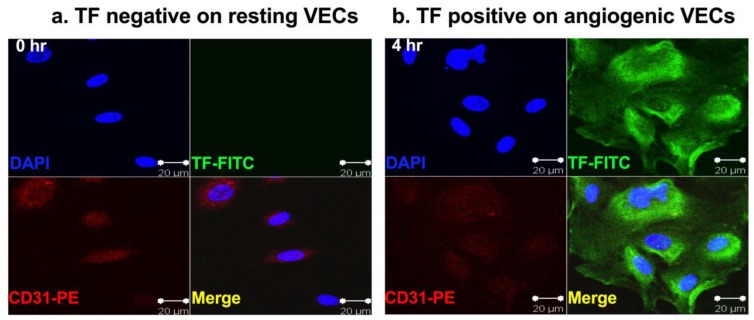
Tissue factor (TF) is an angiogenic specific receptor. Representative confocal imaging of TF (green) and endothelial marker CD31 (red) expression on human microvascular endothelial cells (HMVEC) before 0-hour (0 h) as a normal resting vascular endothelial cell (VEC) model (**a**) and 4 h (4 h) after vascular endothelial growth factor (VEGF) stimulation (4–6 h reaching peak expression) as an angiogenic VEC model (**b**). Cell nuclei were counterstained by DAPI (4′,6-Diamidino-2-Phenylindole, Dihydrochloride) (blue). Scale bars: 20 μm. Modified from ref. [15].

**Figure 2:**
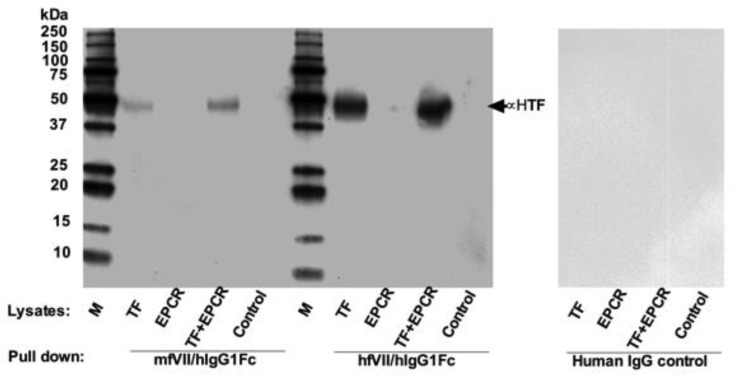
Tissue factor (TF) is the therapeutic target for fVII-targeted immunoconjugate (ICON). Representative Western blots using mouse ICON (mfVII/hIgG1Fc, an immunoconjugate of murine fVII fused to the Fc domain of human IgG1, called an ICON) and human ICON (hfVII/hIgG1Fc, human fVII fused to human IgG1 Fc immunoconjugate) to immune-precipitate their cognate receptor TF that was detected by monoclonal antibody against human TF (HTF) (clone HTF1). Note that both mouse and human ICONs contain a coagulation active site mutation (K341A) in their fVII peptides. The negative controls were untransfected Chinese Hamster Ovary (CHO-K1) cells. Human IgG was an isotype control. Cell lysates were derived from CHO-K1 cells expressing tissue factor (TF), endothelial protein C receptor (EPCR) or both (TF + EPCR). Modified from ref. [15].

**Figure 3:**
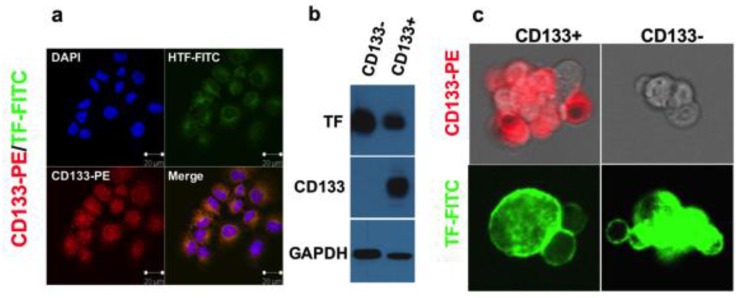
TF is a novel oncotarget in cancer stem cells (CSCs) isolated from *in vitro* cultured human lung cancer H460 cell line (**a**), human triple-negative breast cancer MDA-MB-231 line (**b**) and from patients’ breast tumor tissues (**c**). (**a**) CD133+ CSCs from H460 lung cancer cell line was immunofluorescently stained for expression of CD133 (red) and TF (green). Their nuclei were stained by DAPI (blue) and the cells were photographed under confocal microscopy (Zeiss). Scale bar: 20 μm. (**b**) Immunoblotting for TF expression on CD133+ CSCs and CD133- non-CSC MDA-MB-231 cells. CD133 expression was confirmed on CD133+ CSCs and GAPDH was used for assessing sample loading. (**c**) Representative imaging of TF expression on breast cancer patients’ CD133+ CSCs and CD133-non-CSCs, CD133 expression was confirmed on CD133+ CSCs (Original magnification: 25 μm under ZEO Fluorescent Cell Imager, Bio-Rad, Hercules, CA, USA). Modified from ref. [16].

**Figure 4:**
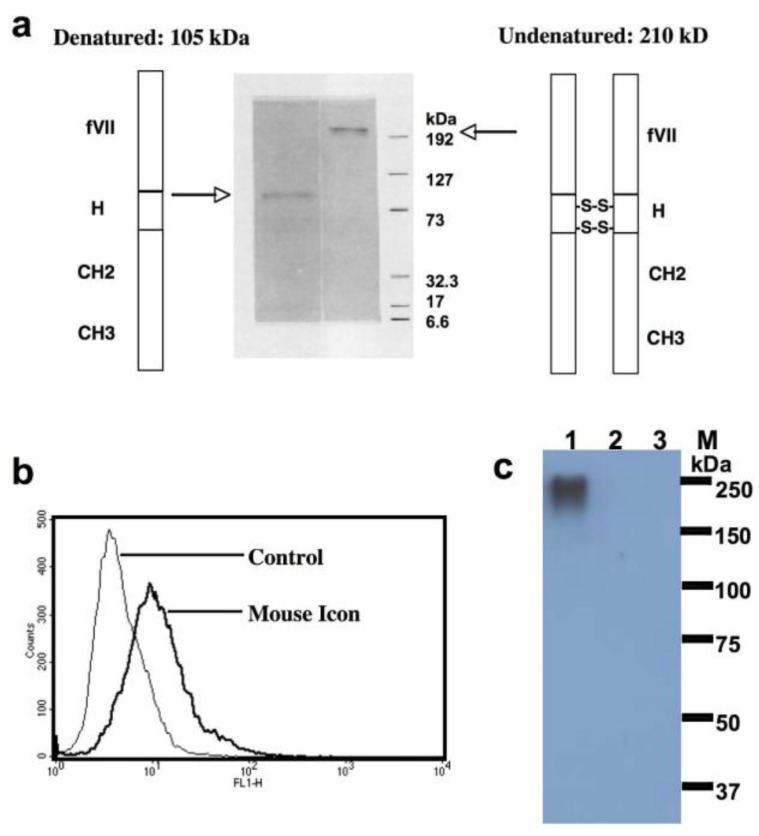
Diagram and characterization of the first generation TF-targeting immunoconjugate (ICON) protein. (**a**) Molecular weight of the ICON protein produced by Chinese Hamster Ovary (CHO) cells analyzed by SDSPAGE. fVII: mouse factor VII with K341A mutation; H: hinge region of a human IgG1 Fc; CH2 and CH3: the second and third domains of the constant region on the heavy chain of a human IgG1 Fc. (**b**) Binding activity of ICON protein to human tongue cancer TCA8113 cells by flow cytometry. Control: TCA8113 cancer cells were not incubated with ICON but with secondary antibody FITC. Mouse ICON: the cells were incubated with ICON protein then with the secondary antibody to human IgG Fc FITC. (**c**) Immunoprecipitation Western-blotting analysis of ICON protein production by TCA8113 cells one day after infection with AdmICON (lane 1) or AdBlank (lane 2). The serum free culture medium from uninfected TCA8113 cells was used as an uninfected control (lane 3). M: Protein markers (Bio-Rad). Molecular weights (kDa) of the protein markers are indicated. Modified from ref [84].

Tissue factor (TF) is a 47-kDa membrane-bound cell surface receptor [1,2,3]. It is also known as thromboplastin, coagulation factor III (fIII) or CD142. Under physiological condition, TF is not expressed by circulating peripheral blood lymphocytes and quiescent vascular endothelial cells (VECs). TF expression is restricted to the cells that are not in direct contact with the blood, such as pericytes, fibroblasts and smooth muscle cells, which are localized in the sub-endothelial vessel wall and sequestered from circulating coagulation factor VII (fVII), the natural ligand for TF. In these cells, the majority of TF is localized in intracellular pools [4]. Upon disruption of vessel wall integrity, TF in pericytes and smooth muscle cells is released and can be bound by fVII, leaking from blood circulation, to initiate blood coagulation in order to stop bleeding [5,6]. Besides its role as the primary initiator of coagulation, TF is also a modulator of pathological angiogenesis [7,8,9]. It is worth noting that there is a truncated version of TF, called alternatively spliced TF (asTF), which lacks the transmembrane and cytoplasmic domains and therefore, is not membrane bound as a soluble isoform. The soluble asTF also plays roles in cancer and angiogenesis [10,11,12,13,14]. However, this review will focus on the membrane-bound TF, also called full length TF (flTF), which is an angiogenic specific receptor since it is selectively expressed on vascular endothelial growth factor (VEGF)-stimulated human microvascular endothelial cells (HMVEC) as an angiogenic VEC model (Figure 1) [15]. TF is also the therapeutic oncotarget for cancer cells and cancer stem cells (CSC) [16] (Figure 2 and Figure 3) for fVII-targeted immunotherapy using coagulation active site-mutated fVII-IgG1 Fc immunoconjugate (called an ICON) (Figure 4) and fVII-targeted photodynamic therapy (fVII-tPDT) using fVII-conjugated photosensitizers) [15,16], as summarized below.

Angiogenesis, the formation of new capillaries from pre-existing vessels, is involved in both physiological conditions (such as reproduction and tissue repair) as well as in more than 20 human diseases [17], including but not limited to cancer [17,18], age-related macular degeneration (AMD), endometriosis and rheumatoid arthritis (RA) [19,20,21]. In cancer, angiogenesis was identified as one of the “hallmarks of cancer” by Hanahan and Weinberg [22,23] due to the recognition that this process is crucial during the transition from benign hyperplastic nodules to malignant lesions [18]. Identification of target molecules specific for angiogenic VEC, the inner layer of pathological neovasculature, is critical for discovery and development of neovascular-targeting therapy for these pathological angiogenesis-dependent, clinically significant human diseases.

## 2. Tissue Factor in Pathological Neovasculature of Cancer, Age-Related Macular Degeneration and Endometriosis

Vascular endothelial growth factor (VEGF) plays a central role in angiogenesis-dependent cancer and non-malignant human diseases [24], such as macular degeneration [25], rheumatoid arthritis [26] and endometriosis [27]. Specifically, VEGF stimulates angiogenesis by binding to VEGF receptors on VECs in the pathological neovasculature (usually micro- or capillary vessels) in those angiogenesis-dependent diseases. It was previously known that VEGF could induce TF expression on human umbilical vein endothelial cells (HUVEC) [10,15,28,29,30], a commonly used VEC model in angiogenesis studies. Noting that although VEGF receptors are relatively expressed at higher levels on tumor VECs, they are also expressed by normal VECs [31], indicating that VEGF receptors are not specific for neovascular endothelial cells. To better mimic pathological angiogenesis, an ideal angiogenic VEC model should be derived from micro- or capillary vessels. Using VEGF-induced *in vitro* angiogenic VEC models, our laboratory recently reported that, unlike VEGFRs, TF is an angiogenic specific receptor and the target for ICON immunotherapy (Figure 4) and fVII-tPDT [15]. We reviewed below its selective expression on angiogenic VECs *in vivo* in the pathological neovasculature of cancer [7,32,33,34,35,36], AMD [19] and endometriosis [21] from animal models to patients.

### 2.1. Tissue Factor Expression in Pathological Neovasculature of Cancer

TF expression on tumor VECs was first reported by Contrino et al. in 1996 in primary tumor tissues from 7 breast cancer patients [32]. Importantly, they also reported that TF expression was not detected in normal VECs in adjacent breast tissues. Hu and Garen independently reported that TF was selectively expressed in tumor neovasculature of human melanoma xenografts *in vitro* and *in vivo* [33,37]. Our laboratory further showed that TF was specifically expressed on the tumor VECs in tumor xenografts of human lung cancer [35] and chemoresistant breast cancer [36] grown in mice but was not expressed on resting VECs in the brain, lungs and spleen of mice [35].

### 2.2. Tissue Factor Expression in the Neovasculature of Age-Related Macular Degeneration

Age-related macular degeneration (AMD) is the leading cause of blindness in the elderly population (age 55 and older) globally. Severe loss of central vision frequently occurs with the exudative (wet) form of AMD, as a result of the formation of a pathological choroidal neovasculature (CNV) that damages the macular region of the retina. To identify a therapeutic target for AMD, in collaboration with the Kaplan laboratory during his tenure at the University of Louisville, Bora et al. reported in 2003 that the endothelial cells of the CNV membrane selectively expressed TF in a pig model [19], whereas the normal retinal vascular endothelium did not express TF. The normal choroidal endothelium also did not express TF [19]. Several earlier studies have shown the presence of growth factors, including FGF, TGF and VEGF in surgically-excised CNV [38,39,40] and tumor necrosis factor α (TNFα) in macrophages in CNV [41]. In 2002, Grossniklaus et al. immunostained post-mortem eyes with CNV and surgically-excised CNV for expression of VEGF and TF [42]. The results [42] showed that VEGF was variably expressed in macrophages and strongly expressed in Retinal pigment epithelium (RPE), a major component of CNV both in post-mortem eyes and surgical specimens. VEGF was also expressed in fibroblasts and photoreceptors. TF was strongly expressed in macrophages and variably expressed in RPE. There was stronger staining for VEGF and TF in inflammatory active versus inflammatory inactive surgically excised CNV [42]. Taken together, these growth factors, including VEGF and TNFα in macrophages and RPE, can contribute to CNV angiogenesis and induction of TF in CNV.

### 2.3. Tissue Factor Expression in the Neovasculature of Endometriosis

Endometriosis is a gynecological disorder characterized by the presence of endometrial tissue, the inner layer of uterus, outside of the uterus. Endometrial lesions are primarily located on the pelvic peritoneum and ovary but can also be located in the pericardium, pleura, lung and even the brain. The disease affects up to 10% of all reproductive-aged women and the prevalence rises to 20%–50% in infertile women. Dr. Lockwood’s laboratory has extensively examined the expression of TF in endometriosis [43,44,45,46]. In normal endometrium, TF expression is limited to stromal cells of the secretory phase with far lower expression in glandular epithelium. In endometriosis, however, TF is greatly overexpressed in both glandular epithelium and stromal cells. Interestingly, the most intense TF immunostaining was observed on macrophages in endometriotic tissues. In 2010, Krikun et al. reported that the endothelial cells in ectopic endometriotic lesions highly expressed TF [21], whereas no TF was detected on gland cells, stromal cells, endothelial cells and vessel walls in eutopic proliferative endometrium from patients [21].

## 3. Tissue Factor Expression in Cancer

### 3.1. Tissue Factor Expression on the Cancer Cells of Solid Cancers, Leukemia and Sarcoma

In addition to its expression on tumor neovasculature, TF is also highly expressed on the cancer cells in solid cancers [47,48,49] and leukemia [49]. As summarized in Table 1, TF expression is detected on the cancer cells in 80%–100% of breast cancer patients, 40%–92% of lung cancer patients and 84% of ovarian cancer patients [49]. Interestingly, Goldin-Lang et al. [12] reported that 8 out of 12 (66.7%) adenocarcinoma lung cancer patients were moderately positive for flTF when using a rabbit polyclonal antibody against flTF (American Diagnostica, Stamford, CT, USA), whereas 11 out of those same 12 tumors (91.7%) were moderately positive for asTF when using a polyclonal rabbit anti-human asTF antibody (vendor not listed). Similar to cancer of the breast, lung and ovary, TF is also expressed at high percentages in many other human solid cancers (Table 1) [16,49], for instance, 95% in primary melanoma and 100% in metastatic melanoma, 53%–90% in pancreatic cancer, 57%–100% in colorectal cancer, 63%–100% in hepatocellular carcinoma, 60%–78% in primary and metastatic prostate cancer and 47%–75% in glioma.

Leukemia is a malignant neoplasm of hematopoietic tissue originating in the bone marrow and infiltrating the peripheral blood and often also the spleen, liver and lymph nodes. Acute leukemia, including acute myeloid leukemia (AML) and acute lymphocytic leukemia (ALL) are characterized by proliferation of immature cells or blasts. If untreated, death usually occurs within 6 months in most cases. ALL is the most common childhood malignancy and the second most common adult leukemia and AML is the second most common childhood malignancy. It was reported that TF is expressed on the human leukemic cell lines HL-60 [72,73,79,85,86], Molt-4 [87], THP-1 [11,87] and on leukemic cells from patients with AML [73,74,75,76,77,78] and ALL [79,80]. TF is not expressed on the normal peripheral mononuclear cells unless stimulated by endotoxin or other cytokines [72], nor on myeloid precursor cells [75]. TF was also detected in the plasma of patients with leukemia [79,80] and in HL-60 culture medium [79].

In sarcoma, TF expression was also detected on mouse Meth-A sarcoma cells [81], rat osteosarcoma cells [82] and vascular origin of Kaposi’s sarcoma [83]. It remains to investigate if TF is expressed in human sarcoma.

### 3.2. Tissue Factor Expression on Cancer Stem Cells

Besides the cancer cells and tumor neovasculature, cancer stem cell (CSC) is also an important tumor compartment in the tumor microenvironment. CSC contributes to tumor angiogenesis, resistance to multiple therapies [88,89] and metastasis [88,90,91]. Targeting CSC therapy can treat cancer at the root and may overcome drug resistance, recurrence and metastasis. Our group recently reported, to our knowledge for the first time, that TF is also expressed on CD133+ and CD24-CD44+ cancer-initiating stem cells and TF can serve as a novel oncotarget for CSC isolated from human cancer cell lines (such as breast, lung, ovarian, head and neck cancer), tumor xenografts and breast cancer patients [16]. Furthermore, TF-targeting immunotherapy agent ICON (discussed below) can eradicate CSC without drug resistance [16].

## 4. Tissue Factor in Rheumatoid Arthritis

Rheumatoid arthritis (RA) is a chronic, often progressive, systemic inflammatory condition of unknown cause. It is characterized by a mononuclear infiltration (T cells, B cells, plasma cells and macrophages) into the synovial tissue and a symmetric, erosive arthritis of peripheral joints but it may also cause systemic manifestations. Tumor necrosis factor α (TNFα) plays an important role in the pathogenesis of RA [92].

### 4.1. TF Expression in Arthritic Joints

Busso et al. [93] immunohistochemically stained synovial tissue specimens from 10 RA patients and reported that TF expression was detected in fibroblasts, smooth muscle cells and macrophages but not in endothelial cells. Chen et al. [94] observed TF expression on Ki-67 positive synoviocytes, B cells and endothelial cells. The controversial results regarding TF expression on endothelial cells in RA could be due to the time point at which TF expression was evaluated. We hypothesize that upon stimulation of pro-inflammatory cytokines and growth factors, endothelial cells express TF in the early stage of RA (acute phase, for example, TF reaching peak expression at 4–6 h post VEGF stimulation, Figure 1b) and then endothelial TF expression may decrease or even disappear in later stages of RA (chronic phase, for example, TF expression started decreasing 8–24 h post VEGF stimulation) (Figure 1 in reference [15], not shown here). Nevertheless, the two published studies provided independent evidence supporting our hypothesis that TF is expressed by macrophages, B cells, Ki-67 positive synoviocytes and angiogenic VECs in RA and targeting TF represents a novel therapeutic approach for immunotherapy of RA. However, it remains to investigate what role TF plays on each of synovial cells in the initiation and progression of RA and if other cytokines also contribute to induction of TF on those synoviocytes.

### 4.2. Angiogenesis and Angiogenic Endothelial TF in RA

RA is also associated with angiogenesis, which enables leukocyte transendothelial migration into the inflamed synovial tissue [17,95,96,97,98,99,100,101,102,103,104]. There are numerous angiogenic mediators, such as TNFα and VEGF and endogenous inhibitors in the RA synovium with an imbalance yielding to increased capillary formation in arthritis. Specifically, vascular endothelial cells (VECs) are involved in a number of mechanisms underlying synovial inflammation [105]. Angiogenic VECs are responsible for increased vascular permeability, leukocyte extravasation (a key feature of inflammation) and secretion of numerous inflammatory mediators during the initiation and progression of RA. Anti-angiogenesis has also been tested for treatment of RA [95]. Many pro-inflammatory cytokines and growth factors such as TNFα, IL-1 and VEGF are known stimuli for induction of TF on VECs [106]. Thus, angiogenic VECs can serve as a target for TF-targeting therapy of RA.

### 4.3. Macrophages in RA Expressing TF

It is well documented that macrophages play several roles in RA initiation and progression. First, macrophages can serve as one of the antigen presenting cells to abnormally present self-antigen leading to activation of autoreactive T cells. Second, macrophages produce and secrete pro-inflammatory cytokines, chemokines, growth factors and enzymes, such as TNFα, IL-1, IL-6, IL-18, IL-15 and IL-32, to further activate other cells, contributing to disease progression. Third, macrophages stimulate synoviocytes to release enzymes, such as collagenases and proteases, which may lead to cartilage and bone damage. We believe targeting macrophage represents a novel therapeutic approach for the treatment of RA. It has been documented that TF is expressed by macrophages in rheumatoid synovium [93,94]. Importantly, TF is not normally expressed by unstimulated monocytes [107,108] but TF can be induced on monocytes by inflammatory mediators including bacterial lipopolysaccharide (LPS, also known as endotoxin) [109], TNFα [110] and IL-1 [111].

### 4.4. Fibroblasts in RA Expressing TF

It is documented that TF is expressed on human fibroblast lines [112,113] and human embryonic fibroblasts [114]. Synovial fibroblasts are involved in the pathogenesis of RA via secreting a wide range of cytokines, chemokines, growth factors and enzymes such as matrix metalloproteinases (MMPs). Studies have shown that inhibiting the growth of synovial fibroblasts could reduce the severity of inflammatory arthritis [115]. Thus, targeting fibroblast via binding to TF may lead to development of novel therapeutic agents for the treatment of RA.

### 4.5. B Cells in RA Expressing TF

B cells are another type of infiltrating immune cells in arthritic joints in RA. B cells play an important role in the pathogenesis of RA, not only serving as the precursors of auto-antibody producing plasma cells but also being involved in antigen presentation, T cell activation and cytokine production [116]. Thus, B cell-directed therapy may provide therapeutic effect in the treatment of RA [117,118,119]. A recent study showed that B cells in human RA express TF [94], whereas normal B cells do not express TF [120]. The reason why RA-associated B cells express TF is still unknown. It could be due to induction by one or a mixture of inflammatory cytokines and chemokines. As evidence, a subpopulation (CD19+CD79b+CD38+CD40+CD5-) of normal human B cells, representing 30% of total B cells, expressed TF after induction by phorbol myristate acetate (PMA) [120,121]. Interestingly, T cells and natural killer (NK) cells do not express TF even after stimulation via LPS or PMA [120]. We previously observed that the NK cell is the major effector cell to mediate antibody-dependent cell-mediated cytotoxicity (ADCC) effect of TF-targeting ICON immunotherapy *in vitro* and *in vivo* in an animal model of cancer [84]. The finding of negative TF expression on NK cells is very important not only to better understand the efficacy but also to ensure the safety of TF-targeting immunoconjugates in clinical trials.

## 5. Cytokines and Growth Factors in RA, Endometriosis and Tumor Microenvironment Contributing to Induction of TF and Angiogenesis

Many cytokines and chemokines are present in rheumatoid synovium [122] and/or in the plasma of RA patients [123,124,125], including pro-inflammatory cytokines (e.g., IL-1, IL-6, TNFα, IL-12, IL-15, IL-17, IL-18, IFNγ, GM-CSF, etc.), anti-inflammatory cytokines (IL-10, IL-1Rα, TGFβ, IL-11, IL-13, etc.), chemokines (e.g., IL-8, MIP-1α, MCP-1, RANTES, etc.) and growth factors (e.g., VEGF, PDGF, FGF). Some of these stimuli can contribute to angiogenesis and increased vascular permeability of VECs (e.g., VEGF) [26] and/or to induction of TF on VECs (e.g., TNFα) [126] or on monocytes (LPS) [109], TNFα [110] and IL-1 [111].

Due to the scope of this review, we did not discuss and summarize all growth factors and cytokines commonly involved in RA, endometriosis, AMD and tumor microenvironment. As discussed above, however, VEGF, a potent growth factor, plays a central role in angiogenesis-dependent cancer and non-malignant human diseases [24], such as AMD [25], RA [26] and endometriosis [27].

## 6. Tissue Factor in Macrophage-Involved Human Diseases

### 6.1. Tissue Factor in Atherosclerosis

Atherosclerosis is an inflammatory disease characterized by the accumulation of lipids in medium to large sized arteries, such as coronary arteries. During atherosclerosis, formation of atherosclerotic plaques in the vessel wall results in narrowing of the lumen of the artery. Atherosclerosis and subsequent atherothrombosis is the leading cause of death in the world. Atherosclerotic plaques are highly procoagulant largely due to the high levels of TF [127,128,129], which is expressed on macrophages and vascular smooth muscle cells in the plaques as well as on microvesicles (also known as microparticles or extracellular vesicles) and foam cell-derived debris within the necrotic core (see the review by Tatsumi and Mackman) [130]. It is worth noticing that normal monocytes do not express TF [107,108]. Interestingly, over 90% of microvesicles within plaques are CD14 positive [131], suggesting their origin of monocyte/macrophage. Several groups [132,133,134,135] have reviewed and linked TF to atherothrombosis and atherosclerosis. Animal models of atherosclerosis have been developed in mice, rabbits, swine and non-human primates, of which mice and rabbits are the most commonly used models. Importantly, similar to the atherosclerosis in humans, high levels of TF are also present in atherosclerotic lesions in rabbit models and in the *Apoe*^−/−^ mouse model [130]. The findings of TF expression in these animal models are very important. This is because it provides not only animal models mimicking the progression of atherosclerosis in humans for basic science research but also provides animal models for testing TF-targeting therapeutic agents for the treatment of atherosclerosis in humans. In addition, hypercholesterolemia [136] and smoking [137] can increase the levels of TF-expressing monocytes and TF-positive microvesicles in atherosclerotic lesions, which could be induced by oxidized LDL via engagement of a TLR4/TLR6 complex [138].

### 6.2. Tissue Factor Expression on HIV-Infected Macrophages

Rapidly after the discovery of the human immunodeficiency virus-1 (HIV-1), it was found that HIV-1 has two types of major target cells in peripheral blood in vivo, namely T lymphocytes, which have been extensively studied and macrophages [139,140], which have been neglected but deserve to be extensively investigated based on the observations described below. While the viral replication cycle is usually rapid and cytopathic in T cells, infected macrophages survive for months *in vitro* and *in vivo* and accumulate large vacuoles containing infectious viral particles. As a result, HIV genes are actively expressed and viral particles are assembled in HIV-infected macrophages [139]. Thus, macrophages play a critical role in the pathogenesis of HIV infection for early stage viral transmission and dissemination within the host and more importantly, as a reservoir of virus persistence. In addition, macrophages in chronic HIV infection selectively express a cell membrane receptor TF [141]. However, TF is not normally expressed by unstimulated monocytes [107] and other quiescent blood cells and VECs in blood vessel walls [29,30,33,34,142]. Elevated TF on macrophages contributes to increased risk of *in vivo* coagulation, i.e., arterial and venous thrombosis, a common adverse effect in HIV patients after highly active antiretroviral therapy (HAART) [141]. In addition, the level of macrophage TF was correlated with the HIV level in plasma [141]. TF expression could be induced on monocytes by LPS [141], which is a bacterial product probably derived from the gastrointestinal tract and has high circulating levels in chronically HIV-infected individuals [143]. Thus, HIV-infected macrophages are considered to be a reservoir for spreading the virus and contribute to increased risk of intravascular thrombosis due to TF expression.

### 6.3. Tissue Factor Expression in Ebola-Infected Macrophages

The Ebola virus can cause acute mortality in approximately 80% of outbreaks in humans and nearly 100% in monkey models, due to severe hemorrhagic fever. The mechanism underlining coagulation abnormalities in Ebola hemorrhagic fever is that the Ebola virus can induce TF expression in primate monocytes and macrophages during viral replication [144]. Blockage of fVIIa/TF by a recombinant nematode anticoagulant protein c2 (rNAPc2) reduced the level of TF activity and significantly increased the survival of treated non-human primates in a rhesus macaque model of Ebola hemorrhagic fever [145].

## 7. Targeting TF Antibodies and Antibody-Like Immunoconjugates in Preclinical Studies

### 7.1. First Generation of TF-Targeting Antibody-Like Immunoconjugates (Called an ICON or ICON-1)

In earlier work at Yale University, Zhiwei Hu and Alan Garen co-invented the first neovascular-targeting Immuno-Conjugate named ICON (Figure 4) [33,34,37,84]. ICON is a chimeric antibody-like homodimer with a molecular weight (MW) of 210 kilodalton (kDa) that consists of murine or human factor VII (fVII, the full-length peptide with 406 amino acid residues, the natural ligand to TF) fused to the Fc region of IgG1 (Figure 4a) [33,34,37,84]. The procoagulant effects of ICON-encoded zymogen fVII have been significantly eliminated via targeted mutation of the lysine reside at position 341 (K341A) [34].

ICON can be administered via intravenous injection of a recombinant protein [142] or intra-lesional injection of an adenovirus vector [34,37,84]. Intra-lesional ICON immunotherapy of experimental melanoma, prostate and head and neck tumors leads to marked tumor inhibition and in some cases, complete eradication without affecting normal tissues [33,34,37,84]. Upon binding to TF-expressing malignant cells, ICON can mediate NK-ADCC and complement-dependent cytotoxicity (CDC) as its mechanism of action [84]. Particularly, NK cell level and activity are crucial for the efficacy of ICON *in vivo* in animal models of cancer and potentially for other therapeutic antibodies in cancer patients [84,146]. Based on these observations, we highly recommend that NK cell actual counting and activity should be monitored in cancer patients before and throughout future clinical trials of TF-targeting immunoconjugates [146].

As discussed above, we believe that TF is a common yet selective therapeutic target in cancer for the cancer cells, tumor neovasculature and CSCs and that TF-targeting therapies represent novel therapeutic approaches with the ability to selectively and effectively target and eliminate these three major and important tumor compartments. These findings may now help us understand that our earlier observations of remarkable effects of ICON immunotherapy without recurrence [34], metastasis [33,37] and drug resistance [16], i.e., complete eradication of well-established primary tumors (up to 600 mm^3^) and metastases, were probably achieved by targeting not only the cancer cells and tumor neovasculature [15,34,37] but also cancer stem cells [16] in mouse models of human and murine prostate [34], melanoma [33,37] and head and neck [84] cancer.

As a neovascular-targeting stand-alone immunotherapy agent, ICON has shown efficacy and safety for the treatment of angiogenesis-dependent diseases via eradication of pathological neovascularization in mouse models of cancer [33,34,37,84,142,147], in mouse and pig models of wet-form macular degeneration [19,20] and in a mouse model of human endometriosis [21]. As a novel neovascular-targeting agent, ICON is being tested in a Phase I clinical trial in ocular melanoma patients and a Phase II clinical trial for patients with age-related macular degeneration (AMD, wet form) (NCT01485588). However, ICON may have some potential limitations in future clinical cancer trials when it is administered systemically as intravenous injections of a recombinant protein. First, its large molecular mass (210 kDa, Figure 4) [84] may limit its ability to penetrate into solid tumor tissues. Second, the procoagulation activity in ICON was not completely depleted [34] and may cause coagulation disorder in cancer patients who usually have a hypercoagulation status [148].

### 7.2. Second Generation TF-Targeting Antibody-Like Immunoconjugate (L-ICON1)

To address these limitations, our laboratory has invented a second-generation ICON (US patent application), named L-ICON1 for lighter ICON. L-ICON1 (GenBank accession no. KX760097) consists of only the light chain (the first 152 aa) of fVII fused to an IgG1 Fc. L-ICON1 has the following important improvements over the original ICON (Hu et al. 2015 SITC Annual Meeting Abstract and 2016 SITC Annual Meeting Late-breaking Abstract P12), including 50% smaller molecular weight, complete depletion of procoagulation activity and more effective than the original ICON *in vivo* in animal models of cancer.

The ICON and L-ICON molecules have several important advantages as compared to anti-TF monoclonal antibodies and antibody-drug conjugates: (i) The dissociation constant (Kd) for fVII binding to TF is up to 10^−12^ M [149], in contrast to anti-TF antibodies that have a Kd in a range of 10^−8^ to 10^−9^ M for TF [150]. (ii) ICON is produced by recombinant DNA technology, allowing mouse ICON (mouse fVII/hIgG1 Fc, GenBank accession no. AF272773) to be made and tested in animal models of diseases and human ICON (human fVII/hIgG1 Fc, also named hI-con1 or ICON-1, GenBank accession no. AF272774) to be made from human sources for future clinical trials without the need of a humanization process that is required for monoclonal antibodies. (iii) Most of antibody-drug conjugates (ADCs) exist as heterogeneous mixtures and require sophisticated site-specific conjugation technologies [151].

### 7.3. TF-Targeting Antibodies and Antibody-Drug Conjugates (ADC)

Several TF-targeting humanized monoclonal antibodies and/or antibody-drug conjugates (TF-ADC) are being studied and reviewed in preclinical and clinical studies [10,152,153,154]. Breij et al. generated humanized IgG1 antibodies (HuMab) against TF in humanized mice using a purified peptide of extracellular domain of TF and TF-expressing NSO cells [152]. Three of them, named TF-011, -098 and -111, could induce efficient inhibition of TF:fVII-dependent intracellular signaling, ADCC and rapid receptor internalization but had minimal impact on TF procoagulant activity *in vitro*. They conjugated those TF HuMab clones with cytotoxic agents and showed that HuMax-TF-ADC was the most potent ADC and the dominant mechanism of action *in vivo* was auristatin-mediated tumor cell killing. TF-011-MMAE induced complete tumor regression in patient-derived xenograft (PDX) models with variable levels of TF expression. Interestingly, the TF-targeting ADC was also effective in the PDX models with TF expression in 25% to 50% of their tumor cells. We believe the reason for the efficacy of the ADC, even in a low TF expressing tumor cell model, is that in addition to cancer cells, the TF-targeting ADC might also target other TF-positive tumor compartments, such as tumor neovasculature and/or cancer stem cells that selectively express or overexpress TF and could be targeted and eradicated by TF-targeting ICON immunotherapy and fVII-tDT *in vitro* [15,16] and *in vivo* [33,37]. The results of ADC demonstrated independently that TF-targeting immunotherapy could have a therapeutic potential to treat multiple types of solid cancers, even with low levels of TF expression on their tumor cells. They further compared the efficacy of TF-targeting ADC with those targeting other cancer cell receptors, such as EGFR and HER2 [155]. They conjugated TF, EGFR and HER2-specific antibodies with duostatin-3, a toxin that induces potent cytotoxicity upon antibody-mediated internalization. They showed that TF-ADC was relatively potent in reducing tumor growth compared with EGFR- and HER2-ADCs in xenograft mouse models.

## 8. TF-Targeting ICON and ADC in Clinical Trials

### 8.1. ICON in Clinical Trials in Patients with Ocular Melanoma and AMD

As a neovascular-targeting agent, ICON has entered clinical trials in patients with AMD and ocular melanoma. A completed Phase 1/2 trial (Clinical trial identifier: NCT01485588) of human ICON (hI-con1, Iconic Therapeutics, South San Francisco, CA, USA) was designed to investigate the safety and tolerability of single and repeated doses of hI-con1™ following administration by intravitreal injection in patients with AMD. The completed multi-center clinical study evaluated the safety and tolerability of a single, intravitreal injection of three different doses of hI-con1 in a total of 18 wet AMD patients. The results have been presented at the American Academy of Ophthalmology Retina Subspecialty Day on 10 November 2012 in Chicago. The results showed that hI-con1 was well tolerated by all patients. Importantly, there were clear indications of dose-related, biologic activity in a number of patients, as indicated by increased visual acuity, reduced retinal thickness and CNV regression [156].

Human ICON was further tested in a Phase 2 randomized, double-masked, multicenter, active-controlled study evaluating administration of repeated intravitreal doses of hI-con1™ in patients with choroidal neovascularization secondary to AMD (NCT02358889). The purpose of this study was to evaluate the safety, biological activity and pharmacodynamic effect of repeated intravitreal doses of 0.3 mg hI-con1 (ICON-1) administered as monotherapy and in combination with 0.5 mg anti-VEGF antibody ranibizumab (Lucentis, Genentech, South San Francisco, CA, USA) compared to ranibizumab 0.5 mg monotherapy in treating 88 patients with choroidal neovascularization (CNV) secondary to AMD. Ranibizumab is a humanized recombinant monoclonal antibody fragment (antigen binding fragment, Fab) that can bind and neutralize human VEGF-A, thereby preventing binding of VEGF-A to its receptors VEGFR-1 and VEGFR-2. The results of the Phase 2a trial was recently presented for the first time at the Angiogenesis, Exudation and Degeneration meeting on 11 February 2017 (ARVO 2017 Annual Meeting Abstracts). No serious ocular adverse events were reported. Repeated intravitreal ICON-1 0.3 mg injections alone or in combination with ranibizumab were well tolerated. And the results provided biological signals of ICON-1 activity on the reduction of CNV progression [157].

Human ICON is also being tested in a Phase 1 trial in patients with uveal melanoma (NCT02771340). The purpose of this multicenter study is to evaluate the safety, tolerability, biological activity, pharmacokinetics and pharmacodynamic activity of single and repeated escalating intravitreal doses (a single or two intravitreal doses of ICON-1 0.3 mg or 0.6 mg) of ICON-1 (hI-con1^TM^) in patients with primary uveal melanoma who are planned to undergo enucleation or brachytherapy of the affected eye. According to the ClinicalTrials.gov website, patient recruitment was completed but the results of this clinical trial are not available yet.

### 8.2. ADC in Clinical Trials in Cancer Patients

An anti-human TF antibody-drug conjugate (HuMax-TF-ADC or called tisotumab vedotin, Genmab, Copenhagen, Denmark) was planned in Phase 1/2 trials (NCT02001623, recruiting and NCT02552121, active but not recruiting) in patients with solid tumors in ovary, cervix, endometrium, prostate, esophagus, lung or head and neck cancers. According to the ClinicalTrials.gov website, the Phase 1/2 trial (NCT02001623) is planning to recruit 144 participants. Currently the same ADC is under a new Phase 2 trial (NCT03245736) in 25 patients with solid tumors known to express TF, including cancers of the ovary, cervix, endometrium, bladder, prostate, esophagus, lung (non-small cell lung cancer, NSCLC) and head and neck (squamous cell carcinoma). The purpose of the trial is to evaluate efficacy and safety of continued treatment with tisotumab vedotin. According to the c website, these clinical studies (NCT02001623 and NCT03245736) are anticipated to be completed in December 2018 and January 2022.

## 9. Conclusions

In summary, TF is expressed on the diseased cells in angiogenesis-dependent human diseases as well as in macrophage-associated human diseases. In angiogenesis-dependent diseases, notably solid cancers, AMD, endometriosis and RA, TF is selectively expressed on angiogenic VECs in the pathological neovasculature. In cancer, TF is also overexpressed by cancer stem cells and by the cancer cells, including solid cancer cells, AML and ALL leukemic cells and sarcoma cells. In RA, TF is additionally expressed by macrophages, B cells, fibroblasts and Ki-67 positive synoviocytes in arthritic joints. In macrophage-associated human diseases, TF is abnormally expressed by monocyte-derived macrophages and foam cells in atherosclerosis and by HIV- and Ebola-infected macrophages in these viral infections. These TF-expressing cells (angiogenic VECs, cancer cells, CSCs, macrophages/foam cells, fibroblasts, B cells) are all involved in disease progression, whereas normal VECs, monocytes, T and NK cells do not express TF. Thus, targeting TF represents novel therapeutic approaches with potential to broadly treat these clinically significant diseases.

As discussed above, there are two approaches for making therapeutic antibodies against TF. One approach was to fuse fVII or its light chain, the natural ligand for TF, to an IgG1 Fc to make ICON and L-ICON1. The other approach was to make humanized monoclonal antibodies against TF. As discussed above, we believe that fVII-containing antibody-like immunoconjugates (ICON and L-ICONs) have advantages over humanized monoclonal antibodies, for higher affinity to TF and no need of humanization. In ADC, those antibodies against TF serve more like a targeting molecule to deliver cytotoxic agents into cancer cells via internalization upon antibody/antigen binding, rather than therapeutic antibodies via ADCC and CDC. The ADC approach is similar to that of fVII-targeted photodynamic therapy that we developed earlier [49], in which fVII (with a coagulation active site mutation K341A) serves as a targeting molecule to selectively deliver photosensitizers into TF-expressing cancer cells [29,30,35,36], tumor VECs [15,29,30,35,36] and CSCs [15] via internalization (reaching peak internalization at 30 minutes post fVII binding to TF) [29].

Some TF-targeting agents, such as ICON and therapeutic ADCs, are being evaluated in early clinical trials, while newer and improved ICONs are being evaluated in preclinical studies with potential to translate into clinical trials. An ideal feature for any TF-targeting antibody-like immunoconjugates or antibodies is that they bind TF but do not have procoagulation activity, so that they will not cause disseminated intravascular coagulation disorders in these human diseases. In this regard, we anticipate that the newer and improved ICONs are more ideal since their procoagulation activity has been completely depleted. Nevertheless, if efficacy and safety of any of these TF-targeting immunoconjugates (ICON and L-ICON1) can be proven in clinical trials, they may impact the treatment regimen for these angiogenesis-dependent as well as macrophage-associated human diseases in the near future.

## Figures and Tables

**Table 1:** Tissue factor expression in solid cancers, leukemia and sarcoma.

Type of Tumor	Case Number	% on TC	% on TVEC	References
Breast cancer	115	81%	ND	[50]
	7	100%	100%	[32]
	213	91%	98.6% (stromal cells)	[51]
	Human chemoresistant breast tumor xenograft from mice *	+	+	[36]
Melanoma	41 primary 42 metastatic	95% 100%	ND	[52]
	Human melanoma xenograft from mice *	+	+	[33]
Lung cancer	25	28%	78% (stromal macrophages, VECs)	[53]
	191 (NSCLC)	43%	ND	[54]
	55	80%	ND	[55]
	50	88%	ND	[56]
	12 (snap-frozen adenocarcinoma NSCLC tissues)	66.7% (8/12) moderately positive for flTF and 91.7% (11/12) for asTF ** vs. the overall negative control healthy tissue	ND	[12]
Hepatocellular carcinoma (HCC)	58	100%	ND	[57]
	62	63%	ND	[58]
Pancreatic cancer	55	53%	TF negative in normal pancreas	[59]
	113	88.4%	ND	[60]
	240 (10 normal pancreas 70 intraductal papillary mucinous neoplasms 40 pancreatic intraepithelial neoplasia, 130 resected or metastatic pancreatic adenocarcinomas)	87.9% overall (77% pancreatic intraepithelial neoplasias 91% intraductal papillary mucinous neoplasms 89% pancreatic cancers)	ND (TF negative in normal pancreas)	[61]
Colorectal cancer	67 primary, of which 18 with liver metastasis	46% of primary, 88.9% of liver metastasis	ND	[62]
	100	57.0%	ND	[63]
	50	100%	ND	[64]
Prostate cancer	67	73%	ND	[65]
	73	75.3%	ND	[66]
	32 early stage 22 advanced stage	78% early-stage 60% advanced stage	ND (TF negative in benign prostate gland)	[67]
	Human prostate tumor in mice ***	+	+	[34]
Ovarian cancer	32	84%	ND	[68]
Glioma	44 (10 benign gliomas 14 anaplastic astrocytomas 20 glioblastomas)	75% overall (10% in Grade I-II, 86% in grade III 95% in grade IV)	ND	[69]
	68 (23 glioblastomas 13 anaplastic astrocytomas 32 low-grade astrocytomas)	47% overall (91.3% glioblastomas, 46.2% anaplastic astrocytomas and 15.6% low-grade astrocytomas)	44% overall (73.9% glioblastomas, 53.8% anaplastic astrocytomas, 0% low grade astrocytomas)	[70]
	34 gliomas 5 normal brain tissues	58.8% overall (20% of grade I 43% of grade II, 58% of grade III 90% of grade IV)	ND (TF negative in normal brain tissues)	[71]
Leukemia	Human AML lines and leukemic cells from patients with AML	+	TF negative on the normal peripheral mononuclear cells unless stimulated by endotoxin or other cytokines [72]	[73,74,75,76,77,78]
	Human ALL lines and leukemic cells from patients with ALL	+	TF negative on myeloid precursor cells [75]	[79,80]
Sarcoma	Mouse Meth-A sarcoma cells	+		[81]
	Rat osteosarcoma cells	+		[82]
	Kaposi’s sarcoma ****	+		[83]

Abbreviations: ND, not determined; TC, tumor cells; TVEC, tumor vascular endothelial cells; NSCLC, non-small cell lung cancer; HCC: hepatocellular carcinoma; flTF: full length TF; asTF: alternatively spliced TF; AML: acute myeloid leukemia; ALL: acute lymphocytic leukemia. Score systems in these studies are generally graded as follows: negative (0%), moderately positive (+, <25%), positive (25%–50%), strongly positive (50%–75%) and very strongly positive (>75%). Positive percentages included all cases graded from moderately positive through very strongly positive. Symbols *, Human breast and melanoma tumor xenografts were removed from mice and paraffin or frozen sections were made and immunohistochemically stained for endothelial TF by using a rabbit polyclonal anti-mouse TF antibody [36] and murine fVII/human IgG1 Fc protein (mouse ICON) [33], respectively. **, Only this study examined flTF and asTF. Other studies in Table 1 and elsewhere throughout the entire text examined flTF or simply referred as TF unless specified. ***, Mouse Icon protein was intravenously injected into the SCID mice carrying subcutaneous human prostate tumor xenografts and the bio-distribution of mouse Icon protein was studied by immunofluorescence staining for the human IgG1 Fc of the mouse Icon protein using an FITC-conjugated anti-human IgG antibody [34]. ****, Kaposi’s sarcoma is vascular origin. +, TF expression was positively detected on those cancer cells/cell lines, regardless of the level of TF expression. The table was updated from ref [49].
